# HIV infection, antiretroviral therapy, and measures of endothelial function, inflammation, metabolism, and oxidative stress

**DOI:** 10.1371/journal.pone.0183511

**Published:** 2017-08-17

**Authors:** Andrew Dysangco, Ziyue Liu, James H. Stein, Michael P. Dubé, Samir K. Gupta

**Affiliations:** 1 Division of Infectious Diseases, Indiana University School of Medicine, Indianapolis, Indiana, United States of America; 2 Department of Biostatistics, Indiana University School of Medicine and School of Public Health, Indianapolis, Indiana, United States of America; 3 Division of Cardiovascular Medicine, University of Wisconsin School of Medicine and Public Health, Madison, Wisconsin, United States of America; 4 Division of Infectious Diseases, University of Southern California Keck School of Medicine, Los Angeles, California, United States of America; Rutgers University, UNITED STATES

## Abstract

**Background:**

HIV-infected patients have an increased risk of cardiovascular disease (CVD). Impaired endothelial function is an early risk factor for CVD in the general population. It is presumed that HIV infection is associated with impaired endothelial function, but results have been inconsistent.

**Objectives:**

Our objectives were to determine the relationships between HIV infection, virologic suppression with antiretroviral therapy (ART), in vivo measures of conduit artery and microvascular endothelial function, and circulating biomarkers of pathways associated with CVD.

**Methods:**

We performed a cross-sectional analysis of three prospectively enrolled groups from a single center: 28 were HIV-infected and virologically-suppressed on a regimen of FTC/TDF/EFV (HIV+ART+), 44 were HIV-infected but not on ART (HIV+ART-), and 39 were HIV-uninfected healthy volunteers (HIV-) matched to the HIV+ART- group for age, sex, smoking status, and height. None had diabetes, uncontrolled hypertension, known CVD, or other pro-inflammatory condition. Flow mediated dilation (FMD), nitroglycerin-mediated dilation (NTGMD), reactive hyperemia velocity time integral (RHVTI), and FMD/RHVTI of the brachial artery were measured, as well as circulating biomarkers of systemic inflammation, metabolism, oxidative stress, and endothelial activation.

**Results:**

No significant differences were found amongst the three groups in FMD (P = 0.46), NTGMD (P = 0.42), RHVTI (P = 0.17), and FMD/RHVTI (P = 0.22) in unadjusted comparisons. Adjusted ANOVA models which included brachial artery diameter, demographics, and conventional CVD risk factors did not appreciably change these findings. In pairwise comparisons, the HIV+ART- group had significantly higher soluble tumor necrosis factor receptor II, soluble CD163, β-2 microglobulin, interferon-γ- induced protein-10, tissue inhibitor of metalloproteinase-1, and vascular cell adhesion molecule-1 compared to the other two groups (all p<0.05). Correlates of endothelial function differed between study groups.

**Conclusion:**

Although untreated HIV infection was associated with elevated levels of several biomarkers of inflammation and endothelial activation, we were unable to demonstrate differences in measures of conduit artery and microvascular endothelial function in this study population.

## Introduction

HIV may be an independent risk factor for CV disease (CVD). Individuals with HIV infection have a significantly increased risk of myocardial infarction (MI) and stroke compared to those without HIV [1–3]. With the ageing HIV-infected population, CVD is expected to play an increasing role in morbidity and mortality in these patients [4].

Endothelial dysfunction is an early step in the development of atherosclerosis and CVD [5, 6]. Flow mediated dilation (FMD) of the brachial artery, an *in vivo* measure of conduit artery endothelial function, has been found to be associated with cardiovascular risk factors [7] as well as predictive of future cardiovascular events [8] in the general population. Also, microvascular endothelial function indices, including reactive hyperemia velocity time integral (RHVTI) and shear stress adjusted FMD (FMD/RHVTI), are also strongly associated with cardiovascular risk factors, especially in younger and relatively healthy cohorts [9], and with cardiovascular events [10]. Inflammation, metabolic abnormalities, and oxidative stress have all been linked to endothelial dysfunction [11–14] and are commonly found in those with HIV infection. As such, it is has been assumed that endothelial function is more impaired in those with HIV infection compared to those who are not infected.

Studies to date assessing the contribution of HIV infection to endothelial dysfunction have been confounded by including patients receiving various antiretrovirals which themselves may induce endothelial dysfunction [15–20]. Much of the HIV-uninfected control data in these studies were obtained from convenience sampling and not from prospectively matched cohorts. Thus, it is not clearly known if HIV-infection itself leads to impaired endothelial function measured as FMD. In addition, there is a lack of data of the effects of HIV infection on microvascular endothelial function measures such as RHVTI or FMD/RHVTI. Therefore, we performed a three-group, prospective study comparing both conduit artery and microvascular endothelial function in HIV-infected patients not yet receiving antiretroviral therapy (ART), HIV-infected patients who were all virologically-suppressed on their first regimen of emtricitabine/tenofovir disoproxil fumarate/efavirenz (FTC/TDF/EFV, or Atripla^®^), and a group of HIV-uninfected healthy controls. We also compared amongst these groups a broad array of biomarkers of inflammation, immune activation, metabolism, and oxidative stress and assessed their relationships with endothelial function.

## Methods

### Study participants

These analyses were performed using data from 72 HIV-positive patients and 39 HIV-negative participants enrolled from other studies by our group (ClinicalTrials.gov NCT00796822, NCT00864916, NCT00919724, and NCT01270802). All participants provided written, informed consent to have their sera samples stored and made available for future analysis. All participants were above 18 years of age and recruited from the HIV outpatient clinics associated with the Indiana University Health medical system.

We defined the following three study groups for these analyses: those with HIV and not receiving antiretroviral treatment (HIV+ART-), those with HIV and receiving virologically suppressive antiretroviral treatment (HIV+ART+), and healthy volunteers without HIV (HIV-). The HIV+ART- group had 45 participants, had been off ART for at least 6 months, and were enrolled in two separate randomized trials assessing the utility of pentoxifylline as an anti-inflammatory agent to improve FMD [21, 22]. We used the baseline data collected prior to pentoxifylline and/or ART initiation for the comparisons in the current study. The HIV- group included 45 participants with no medical co-morbidities and who were prospectively enrolled and matched 1:1 to the HIV+ ART- group based on age (±10 years), sex, height (± 4 inches), and smoking status (current vs not current). These matching criteria were based on factors known to be associated with FMD. Our unpublished data suggested that height is an easily measurable surrogate of brachial artery diameter, which itself is strongly associated with FMD. One FMD study from the HIV+ART- group and six FMD studies from the HIV- group had poor image quality and were considered unevaluable. Thus, from the initial 45 in each group, the remaining 44 in HIV+ART- and 39 in HIV- groups were included in these analyses.

We also included an external control group of HIV+ART+ patients to compare the effects of virologic suppression on the endothelial function parameters and the biomarkers of interest. The 28 participants in the HIV+ART+ group were all receiving emtricitabine/tenofovir disoproxil fumarate/efavirenz and were enrolled in a randomized trial comparing the effects of continued FTC/TDF/EFV with switch to FTC/TDF plus raltegravir on FMD [23]. These study participants had been receiving FTC/TDF/EFV for at least one year and had HIV-1 RNA levels below 50 copies/mL at both screening and within one to six months prior to screening. The data from the baseline visit prior to randomization were used for the current analyses.

Exclusion criteria in all groups included the following: known CVD, diabetes mellitus, uncontrolled hypertension, use of lipid lowering drugs, thyroid abnormalities, systemic inflammatory disease other than hepatitis B or C coinfection, pregnancy or breastfeeding, creatinine clearance <50mL/min, hemoglobin <9.0 g/dL, alanine or aspartate aminotransferase >3 times upper limit of normal, total bilirubin >2.5 times upper limit of normal, or ongoing fever or active infection/malignancy requiring treatment during the study visit [22, 23].

### Study design

Our primary objective was to determine the effects of HIV infection and virologic suppression on in vivo endothelial function parameters. To address this objective, we performed a cross-sectional analysis comparing FMD, RHVTI, FMD/RHVTI, and nitroglycerin mediated dilation (NTGMD) amongst the three study groups described above.

Our secondary objective was to compare circulating biomarkers of pathways that may be biologically related to endothelial function. These included the following: (1) oxidative stress markers [F2-isoprostane and malondialdehyde (MDA)]; (2) systemic inflammatory markers [interleukin-6 (IL-6), high sensitivity C-reactive protein (hsCRP), soluble tumor necrosis factor-α receptors I and II (sTNFRI and sTNFRII), regulated on activation normal T-cell expressed and secreted (RANTES), monocyte chemotactic protein-1 (MCP-1), interferon-γ- induced protein-10 (IP-10), interleukin-8 (IL-8)]; (3) metabolic markers [homeostasis model assessment–insulin resistance (HOMA-IR), total cholesterol, high density lipoprotein cholesterol (HDL-C), low density lipoprotein cholesterol (LDL-C), triglycerides]; (4) cellular and soluble immune activation markers [circulating percentage of activated CD8 cells (CD3+CD8+CD38+HLA-DR+ percentage), β-2 microglubulin (β2MCG), sCD14, sCD163]; and (5) endothelial activation markers [tissue inhibitor of metalloproteinase-1 (TIMP-1), soluble vascular cell adhesion molecule-1 (sVCAM-1), plasminogen activator inhibitor-1 (PAI-1) antigen, asymmetric dimethyl arginine (ADMA)].

Our tertiary objective was to correlate FMD and FMD/RHVTI in each of the three groups with demographic characteristics, blood pressures, body mass indices, HIV parameters, and the circulating biomarkers listed above.

This study was approved by the Indiana University Institutional Review Board. All participants provided written, informed consent.

### FMD and biomarker measurement

All participants were instructed to fast and refrain from smoking at least 8 hours prior to study procedures. All plasma samples for biomarker measurements were drawn and stored at -80°C, and the biomarkers were then batch analyzed at the completion of their respective trials as previously described [22–24]. In addition, the F2-isoprostane was assessed using an LC-MS/MS analytical method, and MDA as measured by monitoring a controlled thiobarbituric acid reaction followed by fluorometrically measuring the MDA adduct produced. Because of incomplete assay reactions and specimen hemolysis, some biomarker values were not available. Only the following circulating biomarkers had five or more missing values: IL-6 (missing n = 6), β2MCG (missing n = 5), IL-8 (missing n = 5), hsCRP (missing n = 8), and RANTES (missing n = 34). Circulating percentages of activated CD8 cells was not measured in the HIV+ART+ group as this measurement was not part of the separate protocol for this group of participants; cells were not saved to perform this assay post hocThe FMD, RHVTI, and nitroglycerin-mediated dilation (NTGMD) were measured after the stated 8 hour fast. All studies were performed by a single registered vascular ultrasonographer using an Acuson CV70 ultrasound machine as described previously [23, 25]. The images from the HIV+ART- and HIV- groups were sent electronically to the University of Wisconsin Atherosclerosis Imaging Research Program core laboratory for reading by a single and blinded investigator (supervised by J.H.S.), while the images from HIV+ART+ group were interpreted at Indiana University by another blinded investigator (S.K.G.). Both used Access Point Web software (Freeland Systems, Westminster, CO) for the image readings. RHVTI could not be accurately determined in three participants from the HIV+ART- group. NTGMD was not performed primarily for safety concerns in ten participants (six from HIV- group, three from HIV+ART- group, and one from HIV+ART+ group).

### Statistical analysis

Continuous variables were summarized by groups using means and standard deviations and were compared among groups using one-way analysis of variance (ANOVA). Categorical variables were summarized by groups using frequency counts and percentages and were compared using Fisher’s exact test. FMD, RHVTI, FMD/RHVTI, and NTGMD were compared among groups using one-way ANOVA. Multiple linear regressions models with adjustments using four different sets of covariates were then constructed: M1 with baseline brachial artery diameter; M2 with age; sex and race; M3 with total cholesterol, HDL-C, LDL-C, triglycerides, HOMA-IR, smoking, SBP, DBP, and body mass index (BMI) ≥ 25; and M4 with all aforementioned factors. Circulating biomarkers were then compared amongst the study groups using one-way ANOVA and Student’s t-test and adjusted for race given that this factor was found to be unequally distributed. Pairwise comparisons were performed if significant differences were found in the ANOVA testing. Spearman’s correlation coefficients were calculated for FMD and FMD/RHVTI with demographics, blood pressures, BMI, CD4 cell counts, HIV-1 RNA levels, and each circulating biomarker in each of the study groups. Associations between sex, race, and smoking status with FMD and FMD/RHVTI were then assessed using Student’s t-test in the various study groups. Adjustments for multiple comparisons were not performed in order to more liberally identify potential biologically relevant associations. Two-sided P values <0.05 were considered statistically significant. All analyses were performed using SAS 9.4 (SAS Institute, Cary, NC).

## Results

### Study group characteristics

The three study groups were generally well-balanced, as shown in Table 1. There were no significant differences in sex, smoking status, age, height, weight, and BMI. However, there were significantly more participants of black race in the two HIV+ groups than in the HIV- group. Diastolic blood pressures were significantly higher in the HIV+ART+ group compared to the other two study groups. As expected, CD4 cell counts and HIV RNA levels were significantly different amongst the groups.

**Table 1:** Baseline characteristics of the three study groups.

Variable	HIV+ART+ N = 28	HIV+ART- N = 44	HIV- N = 39	p-value
**Male sex, n (%)**	26 (93)	35 (80)	32 (82)	0.36
**Black race, n (%)**	17 (61)	27 (61)	13 (33)	0.02
**Current smoker, n (%)**	16 (57)	22 (50)	21 (54)	0.76
**Age, years (SD)**	37.7 (11.5)	36.6 (10.9)	37.5 (11.6)	0.90
**Height, m (SD)**	1.77 (0.07)	1.75 (0.10)	1.75 (0.08)	0.56
**Weight, kg (SD)**	85.42 (17.21)	81.05 (16.31)	83.94 (15.41)	0.58
**BMI, kg/m**^**2**^ **(SD)**	27.28 (5.11)	26.50 (5.13)	27.69 (5.82)	0.63
**Systolic blood pressure, mm Hg (SD)**	125.73 (12.05)	117.51 (15.02)	121.40 (15.07)	0.07
**Diastolic blood pressure, mm Hg (SD)**	79.20 (8.55)	71.77 (8.55)	72.88 (9.11)	<0.01
**CD4 cell count/μL (SD)**	683 (361)	421 (243)	964 (377)	<0.01
**HIV RNA level, log**_**10**_**copies/mL (SD)**	1.46 (1.33)	5.17 (5.43)	—	<0.01

Note: Data presented as number (%) or mean (standard deviation)

ART, antiretroviral therapy; BMI, body mass index

### Effects of HIV infection and virologic suppression on endothelial function

The overall mean (SD) values for FMD, NTGMD, RHVTI, and FMD/RHVTI can be found in Table 2.

**Table 2:** Unadjusted comparisons of endothelial function parameters amongst the three study groups.

Vascular variable	HIV+ART+	HIV+ART-	HIV-	p-Value
**FMD, %**	3.48 (2.54)	3.98 (2.95)	3.24 (2.64)	0.46
**NTGMD, %**	9.05 (8.34)	9.87 (3.76)	10.94 (4.66)	0.42
**RHVTI, cm**	64.36 (23.56)	67.55 (20.70)	74.18 (22.22)	0.17
**FMD/RHVTI, %/cm**	0.06 (0.06)	0.06 (0.05)	0.05 (0.03)	0.22

Note: Data presented as mean (standard deviation)

FMD, flow mediated dilation; NTGMD, nitroglycerin mediated dilation; RHVTI, reactive hyperemia velocity time integral; FMD/RHVTI, shear stress corrected FMD

We found no significant differences in endothelial function as measured by FMD (P = 0.46), NTGMD (P = 0.42), RHVTI (P = 0.17), and FMD/RHVTI (P = 0.22) in unadjusted comparisons (Table 2). Likewise, no significant differences were found among the three groups after further adjustments in models M1, M2, M3, and M4 (data not shown).

### Comparison of biomarkers among the three groups

The circulating biomarker levels measured in this study are shown in Table 3. In overall comparisons among the three groups, we found significant differences in levels of F2-isoprostanes (P<0.01), sTNFRII (P<0.0001), MCP-1 (P = 0.02), IP-10 (P<0.0001), total cholesterol (P = 0.03), HDL-C (P<0.0001), β2MCG (P<0.0001), sCD14 (P<0.0001), sCD163 (P<0.0001), TIMP-1 (P<0.0001), sVCAM-1 (P<0.0001), and ADMA (P<0.0001).

**Table 3:** Unadjusted comparisons of circulating biomarkers amongst the three study groups.

Biomarker	Group	Mean (SD)	Comparison	P-value
**F2-isoprostanes, pg/mL**			Overall	<0.01
	HIV+ART+	20.27 (11.96)	HIV+ART+ vs. HIV+ART-	N.S.
	HIV+ART-	25.64 (13.50)	HIV+ART- vs. HIV-	N.S.
	HIV-	31.63 (15.44)	**HIV+ART+ vs. HIV-**	**<0.05**
**MDA, nmol/mL**			Overall	0.21
	HIV+ART+	4.09 (0.57)	HIV+ART+ vs. HIV+ART-	N.S.
	HIV+ART-	3.84 (0.58)	HIV+ART- vs. HIV-	N.S.
	HIV-	3.99 (0.60)	HIV+ART+ vs. HIV-	N.S.
**IL-6, pg/mL**			Overall	0.57
	HIV+ART+	1.64 (1.16)	HIV+ART+ vs. HIV+ART-	N.S.
	HIV+ART-	2.82 (2.55)	HIV+ART- vs. HIV-	N.S.
	HIV-	3.29 (9.52)	HIV+ART+ vs. HIV-	N.S.
**hsCRP, mg/L**			Overall	0.57
	HIV+ART+	2.83 (2.36)	HIV+ART+ vs. HIV+ART-	N.S.
	HIV+ART-	4.24 (9.88)	HIV+ART- vs. HIV-	N.S.
	HIV-	2.69 2.85)	HIV+ART+ vs. HIV-	N.S.
**sTNFRI, pg/mL**			Overall	0.26
	HIV+ART+	1129.04 (232.10)	HIV+ART+ vs. HIV+ART-	N.S.
	HIV+ART-	1012.62 (325.56)	HIV+ART- vs. HIV-	N.S.
	HIV-	1117.59 (315.92)	HIV+ART+ vs. HIV-	N.S.
**sTNFRII, pg/mL**			Overall	<0.0001
	HIV+ART+	5327.55 (1247.13)	**HIV+ART+ vs. HIV+ART-**	**<0.05**
	HIV+ART-	8550.48 (3388.05)	**HIV+ART- vs. HIV-**	**<0.05**
	HIV-	5806.56 (1134.79)	HIV+ART+ vs. HIV-	N.S.
**RANTES, pg/mL**			Overall	0.23
	HIV+ART+	73264.31 (81780.86)	HIV+ART+ vs. HIV+ART-	N.S.
	HIV+ART-	67050.72 (95421.43)	HIV+ART- vs. HIV-	N.S.
	HIV-	31860.15 (30535.68)	HIV+ART+ vs. HIV-	N.S.
**MCP-1, pg/mL**			Overall	0.02
	HIV+ART+	207.22 (70.90)	HIV+ART+ vs. HIV+ART-	N.S.
	HIV+ART-	269.91 (135.41)	HIV+ART- vs. HIV-	N.S.
	HIV-	210.44 (100.13)	HIV+ART+ vs. HIV-	N.S.
**IP-10, pg/mL**			Overall	<0.0001
	HIV+ART+	207.08 (104.85)	**HIV+ART+ vs. HIV+ART-**	**<0.05**
	HIV+ART-	520.55 (284.60)	**HIV+ART- vs. HIV-**	**<0.05**
	HIV-	150.54 (89.78)	HIV+ART+ vs. HIV-	N.S.
**IL-8, pg/mL**			Overall	0.36
	HIV+ART+	2.49 (1.97)	HIV+ART+ vs. HIV+ART-	N.S.
	HIV+ART-	3.27 (2.01)	HIV+ART- vs. HIV-	N.S.
	HIV-	2.87 (2.60)	HIV+ART+ vs. HIV-	N.S.
**HOMA-IR**			Overall	0.30
	HIV+ART+	1.71 (1.15)	HIV+ART+ vs. HIV+ART-	N.S.
	HIV+ART-	2.17 (2.57)	HIV+ART- vs. HIV-	N.S.
	HIV-	2.32 (2.39)	HIV+ART+ vs. HIV-	N.S.
**Total cholesterol, mg/dL**			Overall	0.03
	HIV+ART+	153.75 (34.86)	HIV+ART+ vs. HIV+ART-	N.S.
	HIV+ART-	142.95 (28.73)	**HIV+ART- vs. HIV-**	**<0.05**
	HIV-	162.92 (32.97)	HIV+ART+ vs. HIV-	N.S.
**HDL-C, mg/dL**			Overall	<0.0001
	HIV+ART+	41.07 (10.77)	HIV+ART+ vs. HIV+ART-	N.S.
	HIV+ART-	37.02 (10.27)	**HIV+ART- vs. HIV-**	**<0.05**
	HIV-	49.84 (18.68)	**HIV+ART+ vs. HIV-**	**<0.05**
**LDL-C, mg/dL**			Overall	0.55
	HIV+ART+	88.91 (34.00)	HIV+ART+ vs. HIV+ART-	N.S.
	HIV+ART-	86.14 (26.61)	HIV+ART- vs. HIV-	N.S.
	HIV-	95.09 (32.31)	HIV+ART+ vs. HIV-	N.S.
**Triglycerides, mg/dL**			Overall	0.15
	HIV+ART+	118.39 (67.03)	HIV+ART+ vs. HIV+ART-	N.S.
	HIV+ART-	99.34 (51.40)	HIV+ART- vs. HIV-	N.S.
	HIV-	90.18 (64.36)	HIV+ART+ vs. HIV-	N.S.
**CD3+CD8+CD38+HLA-DR+, %**			Overall	<0.05
	HIV+ART-	42.30 (16.98)	**HIV+ART- vs. HIV-**	**<0.05**
	HIV-	7.51(5.55)		
**β2MCG, mcg/mL**			Overall	<0.0001
	HIV+ART+	1463163.97 (690501.73)	**HIV+ART+ vs. HIV+ART-**	**<0.05**
	HIV+ART-	3751961.36 (1559440.88)	**HIV+ART- vs. HIV-**	**<0.05**
	HIV-	1942211.72 (549424.29)	HIV+ART+ vs. HIV-	N.S.
**sCD14, ng/mL**			Overall	<0.0001
	HIV+ART+	2454.49 (336.95)	**HIV+ART+ vs. HIV+ART-**	**<0.05**
	HIV+ART-	1964.40 (561.55)	HIV+ART- vs. HIV-	N.S.
	HIV-	1883.96 (285.53)	**HIV+ART+ vs. HIV-**	**<0.05**
**sCD163, ng/mL**			Overall	<0.0001
	HIV+ART+	579.47 (223.35)	**HIV+ART+ vs. HIV+ART-**	**<0.05**
	HIV+ART-	790.15 (255.39)	**HIV+ART- vs. HIV-**	**<0.05**
	HIV-	482.75 (152.62)	HIV+ART+ vs. HIV-	N.S.
**TIMP-1,ng/mL**			Overall	<0.0001
	HIV+ART+	95.34 (16.05)	**HIV+ART+ vs. HIV+ART-**	**<0.05**
	HIV+ART-	116.87 (33.43)	**HIV+ART- vs. HIV-**	**<0.05**
	HIV-	82.72 (20.00)	HIV+ART+ vs. HIV-	N.S.
**sVCAM-1, ng/mL**			Overall	<0.0001
	HIV+ART+	603.00 (154.17)	**HIV+ART+ vs. HIV+ART-**	**<0.05**
	HIV+ART-	1101.63 (356.45)	**HIV+ART- vs. HIV-**	**<0.05**
	HIV-	557.75 (139.95)	HIV+ART+ vs. HIV-	N.S.
**PAI-1, ng/mL**			Overall	0.39
	HIV+ART+	31.65 (18.77)	HIV+ART+ vs. HIV+ART-	N.S.
	HIV+ART-	26.28 (20.82)	HIV+ART- vs. HIV-	N.S.
	HIV-	30.65 (18.63)	HIV+ART+ vs. HIV-	N.S.
**ADMA, μmol/L**			Overall	<0.0001
	HIV+ART+	0.48 (0.09)	**HIV+ART+ vs. HIV+ART-**	**<0.05**
	HIV+ART-	0.62 (0.17)	HIV+ART- vs. HIV-	N.S.
	HIV-	0.60 (0.11)	**HIV+ART+ vs. HIV-**	**<0.05**

Note: Bolded text is for statistically significant comparisons between individual study groups

MDA, malondialdehyde; IL-6, interleukin 6; hsCRP, high sensitivity C-reactive protein; sTNFRI and sTNFRII, soluble tumor necrosis factor receptor I and II; RANTES, regulated on activation normal T-cell expressed and secreted; MCP-1, monocyte chemotactic protein-1; IP-10, interferon-γ induced protein-10; IL-8, interleukin 8; HOMA-IR, homeostasis model assessment–insulin resistance; HDL-C, high density lipoprotein cholesterol; LDL-C, low density lipoprotein cholesterol; β2MCG, beta-2 microglobulin; sCD14, soluble cluster of differentiation 14; sCD163, soluble cluster of differentiation 163; TIMP-1, tissue inhibitor of metalloproteinase-1; sVCAM-1, soluble vascular cell adhesion molecule-1; PAI-1, plasminogen activator inhibitor; ADMA, asymmetric dimethyl arginine; N.S., not significant

We then performed pairwise comparisons of these circulating biomarkers (Table 3). The HIV+ART- group had significantly higher levels of sTNFRII, sCD163, β2MCG, IP-10, TIMP-1, and sVCAM-1 compared to each of the other two groups. In contrast, the HIV+ART+ group had significantly higher sCD14 and lower ADMA levels compared to each of the other groups. The HIV- group had significantly higher HDL-C levels compared to each of the other two groups. F2-isoprostane levels were significantly higher in the HIV- group only when compared to HIV+ART+ group. We found that the HIV+ART- had statistically significantly higher levels of activated CD8 percentages compared to the HIV- group. Although, significantly different in the three-group comparison, MCP-1 was not significantly different in pairwise comparisons between the individual study groups. Adjustment for race in all of these comparisons did not affect these results appreciably.

### Correlates of endothelial function

As shown in Table 4, we found significant correlations (r, p-value) between FMD and both SBP (-0.35, p = 0.02) and DBP (-0.31, p = 0.046) in the HIV+ART- group. FMD was also correlated with sTNFRII (-0.48, p<0.01), IL-8 (0.42, P = 0.03), RANTES (0.48, P = 0.03), and HDL-C (0.41, 0.03) in the HIV+ART+ group. FMD was also correlated with RANTES (0.41, P = 0.049) in the HIV- group. FMD/RHVTI was correlated with sTNFRII (-0.48, P<0.01) and HDL-C (0.38, P<0.05) in the HIV+ART+ group and with IP-10 (0.34, P = 0.03) in the HIV- group. No other correlations, including for age, were found to be statistically significant.

**Table 4:** Statistically significant correlates of endothelial function parameters within the individual study groups.

Group	Factor	FMD	FMD/RHVTI
**HIV+ART-**			
	SBP	-0.35 (p = 0.02)	
	DBP	-0.31 (p = 0.046)	
**HIV+ART+**			
	sTNFRII	-0.48 (p<0.01)	-0.48 (p<0.01)
	IL-8	0.42 (p = 0.03)	N.S.
	RANTES	0.48 (p = 0.03)	N.S.
	HDL-C	0.41 (p = 0.03)	0.38 (p<0.05)
**HIV-**			
	RANTES	0.41 (p = 0.049)	N.S.
	IP-10	N.S.	0.34 (p = 0.03)

Note: Data are presented as correlation coefficient (p-value)

SBP, systolic blood pressure; sTNFRII, soluble tumor necrosis factor receptor II, IL-8, interleukin 8; RANTES, regulated on activation normal T-cell expressed and secreted; IP-10, interferon gamma induced protein 10; FMD, flow mediated dilation; FMD/RHVTI, FMD corrected for shear stress; N.S., not significant

We then assessed in the overall study group and individual study groups other categorical associations with FMD and FMD/RHVTI. We found in the overall group that men compared to women had lower mean (SD) FMD [3.17 (2.18) vs. 5.77 (4.09); p = 0.02] and FMD/RHVTI [0.05 (0.04) vs. 0.09 (0.07); p = 0.03]; however, sex was neither associated with FMD nor FMD/RHVTI in any of the individual study groups. Black race was not associated with either FMD or FMD/RHVTI in the overall study group or in any individual study group. In the HIV+ART+ study group, current smoking vs. no current smoking was associated with higher FMD [4.36 (2.68) vs. 2.31 (1.84); p = 0.03] but not FMD/RHVTI; smoking status was not associated with either vascular parameter in any of the other study groups or overall.

## Discussion

HIV has been associated with increased rates of cardiovascular disease [1–3]. In addition, in vitro studies have demonstrated that HIV particles, including gp140, Nef, and Tat, may damage vascular endothelium [26–30]. In addition, in untreated HIV infection, greater levels of circulating HIV Tat may induce cell surface expression of endothelial leukocyte adhesion molecule-1, VCAM-1, and ICAM-1, which in turns may lead to increased endothelial leukocyte adhesion [31, 32]. It is also possible that specific ART drugs, including protease inhibitors and efavirenz, may increase oxidative stress and, by this mechanism, increase endothelial recruitment of mononuclear cells [33]. Moreover, HIV has been associated with increased systemic inflammation and coagulation, both of which may lead to endothelial dysfunction and are only partly mitigated by ART [34]. For instance, atherosclerosis development may be initiated and accelerated in HIV-infected patients due to increased numbers of activated monocytes which may migrate across the endothelium and form foam cells [35]. Thus, we expected to find both untreated HIV infection and ART-treated HIV to be associated with worse endothelial function. Instead, we unexpectedly did not find that being infected with HIV was related to more impaired physiologic conduit artery or microvascular function as measured using the brachial artery FMD technique.

Previous studies have been mixed on finding impaired FMD when comparing HIV-infected and uninfected persons. Andrade et al [36] found that FMD was significantly reduced in ART-receiving HIV-infected patients compared to those who were untreated and also compared to uninfected controls matched to the HIV-infected patients by age and BMI; however, as was found in our study, there were no significant differences in FMD between the untreated HIV-infected group compared to the uninfected controls. Similarly, Charakida et al [17] and Rios Blanco et al [37] found that in ART-treated children and adults, respectively, that FMD was significantly lower compared to uninfected controls, but that ART-naïve patients did not have significantly impaired FMD compared to uninfected controls. Solages et al [38] and van Wijk et al [39] also both found that treated HIV-infected patients receiving ART had significantly lower FMD compared to uninfected controls, though they did not study specifically HIV-infected patients not receiving ART. However, Nolan et al [40] did not find such differences between treated HIV-infected patients and uninfected controls, findings similar to those in our study. Both Arildsen et al [15] and Oliviero et al [41] found that untreated HIV infection was associated with lower FMD compared to uninfected controls matched for age, sex, and smoking. Thus, it is not clear why there have been such discordant results, but subtle differences in the FMD technique, the study populations, the ART regimens used, and unmeasured confounding variables likely all play a role.

To our knowledge, a comparison of microvascular function using the FMD technique has not previously been performed between HIV-infected and uninfected controls. We found no differences in hyperemic velocity (RHVTI) or sheer stress adjusted FMD (FMD/RHVTI) between the HIV-infected and uninfected study groups. In the only other study to our knowledge that recorded RHVTI in HIV-infected patients, Hatano et al [42] found similar RHVTI values in their HIV-infected patients receiving suppressive ART (68.3 cm) to those found in our study, but they did not compare their results to untreated patients or uninfected controls.

As expected, we found higher circulating levels of several inflammatory biomarkers, including of sTNFRII, sCD163, β2MCG, IP-10, TIMP-1, and sVCAM-1 in the untreated HIV-infected group compared to the other two study groups. However, we found unexpected differences in circulating levels of several biomarkers, specifically F2-isoprostanes and ADMA. We found lower circulating levels of the oxidative stress marker F2-isoprostane in both HIV-infected groups compared to the healthy, uninfected controls. This was unexpected given that HIV infection is presumed to be a pro-oxidant condition. However, previous studies assessing circulating F2-isoprostane levels in HIV did not include uninfected control groups for comparison [43–46]. The other oxidative stress biomarker measured in our study, namely MDA, was not different amongst the three study groups. Thus it is possible in HIV-infected patients with no other major comorbidities and who have not received lipodystrophy-inducing agents, such as those included in our study, there may not be appreciable oxidative stress. It is unclear why though F2-isoprostanes were actually statistically significantly lower in the HIV-infected groups. Thus, our results will, of course, need to be confirmed in larger studies.

We were also surprised to see that ADMA was not elevated in the HIV-infected groups in our study, and in fact, circulating ADMA levels were significantly lower in the HIV+ART+ group compared to the HIV+ART- and HIV- groups. This contrasts to the studies by Beltran et al [47], Jan et al [48], and Haissman et al [49] which found higher ADMA levels in both ART-naïve and treated HIV-infected patients compared to uninfected controls. However, Parikh et al [50] did not find differences in ADMA levels between HIV-infected patients (most of whom were receiving ART) and uninfected controls. We do not know why ADMA levels were lower in our ART-treated HIV-infected patients, but further research in this area certainly will need to be pursued.

When examining correlates of endothelial function, we found that there was heterogeneity based on the HIV status and virologic suppression. In the HIV+ART- group, we actually did not find any correlates of FMD or FMD/RHVTI with any of the biomarkers assessed. The inflammatory biomarker sTNFRII was inversely correlated with FMD and FMD/RHVTI in the HIV+ART+ group only. Conversely, other inflammatory biomarkers, including IL-8 and RANTES were directly correlated with improved FMD in this group. These results contrast with those of Grome et al [51] who found that activated CD8+ cells, but not sTNFRII, were associated with FMD in 70 HIV-infected patients who were similarly treated with FTC/TDF/EFV. We also found that RANTES was significantly correlated with FMD, but not FMD/RHVTI in the HIV- group. HDL-C was also significantly correlated with FMD and FMD/RHVTI in the HIV+ART+ group, but not in the HIV- group. These data suggest that the cardiovascular pathways responsible for endothelial dysfunction may vary depending on HIV status and virologic suppression with ART. However, we do acknowledge that other biomarkers that may be related to cardiovascular disease, but were not measured in this study, may be relevant to assess as potentially contributing to endothelial dysfunction in HIV. For example Zungsontiporn et al [52] found that serum amyloid P was associated with FMD in participants in the Hawaii Aging with HIV-Cardiovascular study.

Notably, we did not find that current CD4 cell count or HIV-1 RNA levels correlated with endothelial function in our study, which agrees with some studies [53–55] but contrasts with other studies that have found higher viral loads being associated with lower FMD [41].

The major strength of the current study was the prospectively matched enrollment of HIV-uninfected study participants to those with untreated HIV infection and the strict exclusion criteria of confounding factors (e.g. no statin use, lack of other co-morbidities); thus, the study groups were fairly homogeneous except for the presence or absence of HIV infection. The imbalances in black race likely did not affect the results given that this variable was not associated with either FMD or FMD/RHVTI and as this variable was adjusted for in our models. In addition, the HIV+ART+ group was quite similar to the other study groups and involved the use of a single ART regimen to limit variability from the potential effects on endothelial function from the use of various antiretroviral drugs. Therefore, our results are less likely to have been affected negatively from unmeasured confounding, though we cannot eliminate this possibility altogether. In addition, both HIV-infected groups were broadly representative of the U.S. HIV population with good representation of both white and black patients with a broad range of both CD4 cell counts and HIV-1 RNA levels. Thus, our results should be externally generalizable to these groups. However, the low numbers of female participants limit generalizability of our results to this specific demographic.

We acknowledge several limitations of this analysis. The sample sizes were relatively small and may thus have been underpowered to find differences in the vascular parameters and circulating biomarker levels amongst the three study groups, although our study was comparable or even larger than many of the studies that did find differences in these parameters. The lack of adjustment for multiple testing may have contributed to false-positive findings. The two different ultrasound readers may have introduced for the comparisons between the HIV+ART+ group and the two other study groups, but the same software, technique, and procedures were utilized for all three study groups. As a cross-sectional analysis, this study could not assess causal relationships. Even though the HIV+ART- and HIV- groups were matched on several key characteristics, the HIV+ART+ group was an external convenience sample; however, mitigating this limitation was that the procedures performed were identical to those in the other study groups and the analyses were adjusted for demographic imbalances. But then we cannot exclude the possibility that different ART regimens may lead to either more impaired or improved FMD results, especially given that we and others have previously reported greater endothelial dysfunction with efavirenz-based regimens [19, 56]. We also acknowledge that if HIV infection is truly associated with endothelial dysfunction compared to those without HIV, then it is possible that the FMD technique used in this study may not be adequately sensitive to find such differences.

In conclusion, patients with either untreated HIV infection or HIV infection controlled by the antiretroviral regimen emtricitabine/tenofovir disoproxil fumarate/efavirenz did not have more impaired physiologic vascular function parameters compared to uninfected healthy volunteers. These results were found despite HIV infection being associated with more deleterious circulating levels of certain biomarkers associated with endothelial dysfunction and cardiovascular disease in the general population, suggesting that endothelial function may depend on differing cardiovascular pathways between those with and without HIV.
